# The Comparative Effectiveness of Traditional Chinese Medicine Exercise Therapies in Elderly People With Mild Cognitive Impairment: A Systematic Review and Network Meta-Analysis

**DOI:** 10.3389/fneur.2022.775190

**Published:** 2022-03-16

**Authors:** Kaiqi Su, Jie Yuan, Huanhuan Liu, Meng Luo, Qi Li, Sutong Liu, Xiaodong Feng

**Affiliations:** ^1^Department of Rehabilitation Medicine, Henan University of Chinese Medicine, Zhengzhou, China; ^2^Rehabilitation Center, The First Affiliated Hospital of Henan University of Chinese Medicine, Zhengzhou, China; ^3^Department of Digestive Diseases, The First Affiliated Hospital of Henan University of Chinese Medicine, Zhengzhou, China

**Keywords:** brain function, exercise therapy, mild cognitive impairment, network meta-analysis, traditional Chinese medicine

## Abstract

**Background:**

Mild cognitive impairment (MCI) in the elderly is a health problem worldwide. Studies have confirmed that traditional Chinese medicine (TCM) exercise therapies can improve MCI. However, which therapy is the best and their impacts on brain function remain controversial and uncertain. This study aims to compare and rank TCM exercise therapies for MCI in the elderly, and analyze their effects on brain function, in order to find an optimal intervention and provide a basis for clinical treatments decision-making.

**Methods:**

The Web of Science, PubMed, EMBASE, Cochrane Central Register of Controlled Trials, Clinical Trials, China National Knowledge Infrastructure (CNKI), Wangfang database, China Science and Technology Journal Database, and Chinese Biomedical Medicine (CBM) were searched through October 28, 2021. Two researchers reviewed all the studies and extracted the data. The ADDIS software version 1.16.8 and the Bayesian hierarchical model were used for pair-wise meta-analysis and network meta-analysis, and the STATA software version 14.0 was used to draw the network evidence plots and funnel plots.

**Results:**

A total of 23 studies on 2282 participants were included in this study. In the pair-wise meta-analysis, TCM exercise therapies (Baduanjin exercise, Tai Chi, Liuzijue exercise and finger exercise) were superior to non-TCM exercise therapies (stretching and toning exercise, usual care, health education and routine daily activities) in terms of MMSE, MoCA and ADL outcomes. In the network meta-analysis, the MMSE outcome ranked Baduanjin exercise (78%) as the best intervention and Tai Chi (36%) as the second. The MoCA outcome ranked Baduanjin exercise (62%) as the best intervention. For the ADL outcome, Baduanjin exercise (60%) ranked the best, and followed by finger exercise (43%).

**Conclusion:**

TCM exercise therapies may improve the cognitive function in elderly patients with MCI. Among the four therapies included, the Baduanjin exercise may be the preferred therapy for MCI in the elderly, and its mechanism may be related to the regulation of cognitive-related brain function and structure.

**Systematic Review Registration:**

https://inplasy.com, identifier: INPLASY202070006.

## Introduction

Mild cognitive impairment (MCI) is a neurodegenerative disease characterized by a decline in learning, memory, executive ability and logical thinking ability, which is defined as a transitional stage of dementia (1–3). In China, the Law on the Protection of the Rights and Interests of the Elderly defines people aged ≥60 years as elderly (4). Studies have demonstrated that the occurrence of MCI is age-related, with a prevalence of 15–20% in the elderly population (5). In addition, the number of patients with cognitive impairment is increasing rapidly each year as the global aging process accelerates (6). During the initial stage of MCI, the daily activities of patients may not be significantly affected. However, without timely intervention, approximately 80–90% of MCI patients will progress to dementia within 5 years (7, 8). It is estimated that by the middle of this century, the number of dementia patients worldwide will reach 115 million, which will place a heavy financial burden on families and society (9).

At present, pharmaceutical interventions of MCI mainly include symptomatic treatments (e.g., vitamin E, acetylcholinesterase inhibitors, galantamine, etc.) and etiological treatments (e.g., nerve regeneration promotion, antihypertensive, antidiabetic, etc.) (10). However, different degrees of side effects and adverse reactions do exist in pharmaceutical interventions. Therefore, non-drug therapy has gradually become the main means of intervention in MCI in recent years, such as autonomic training, cognitive training and exercise therapy. Several studies have concluded that non-drug therapies have a positive impact on hyperlipidemia, hypertension, sleep quality and social networks, thereby modulating patients' psychological and physical comorbidities (11–15).

As an important component of non-drug intervention, traditional Chinese medicine (TCM) exercise therapies are a type of body-mind exercise regimen suitable for all ages base on the holistic concept of TCM and the theory of co-cultivation of body and mind, and have the advantages of being easy to learn, not restricted by venues and equipment, and without adverse reactions. Such as Tai Chi, Baduanjin exercise, Liuzijue exercise and finger exercise, etc. Studies have found that TCM exercise therapies can improve cardiopulmonary function, enhance blood circulation, improve sleep quality and cognitive function, and have been widely promoted worldwide in recent years (16–19). Meanwhile, a systematic review and meta-analysis showed that Chinese mind-body exercises (including Qigong and Tai Chi) can improve the executive function of middle-aged and older adults (20).

However, differences of these therapies in the form, duration, and applicable population are existing. It is unclear which therapy is more appropriate for elderly MCI patients. Although a previously published network meta-analysis has confirmed Baduanjin exercise as the preferred exercise therapy for patients with cognitive impairment (21). However, this study only included articles up to December 2019 and compared the efficacy of Baduanjin exercise, Tai Chi, Liuzijue exercise and Qigong only, while only one outcome indicator was used for probability ranking, which has some limitations. Besides, with the increase in functional magnetic resonance imaging-related studies, MCI has been proved to be closely associated with changes in brain function, while the effect of TCM exercise therapies on brain function is still unclear. Therefore, this study aims to compare and rank the TCM exercise therapies using a variety of different outcomes through a network meta-analysis, and to analyze their impacts on brain function, so as to obtain the optimal treatment plan for elderly MCI patients and provide a basis for clinical treatments decision-making.

## Methods

### Search Strategy

A comprehensive systematic searching was conducted to collect eligible articles in the following databases: Web of Science, PubMed, EMBASE, Cochrane Central Register of Controlled Trials, Clinical Trials, China National Knowledge Infrastructure (CNKI), Wangfang Database, China Science and Technology Journal Database and Chinese Biomedical Medicine (CBM). We established the search strategy which included medical subject headings and free words associated with TCM exercise therapies or MCI. The medical subject headings included “exercise therapy”, “Tai Chi”, “Baduanjin”, “Qi gong”, “Liuzijue”, “finger exercise”, “Wuqinxi”, “five animal exercise”, “Yijinjing”, “classics of tendon changing”, “mild cognitive impairment” and “randomized controlled trials”. The languages were confined to English and Chinese, and the retrieval time was set to the date of database onset to October 28, 2021. Furthermore, we also reviewed the references of the included literature to find additional eligible studies. The detailed search strategies are presented in Supplementary Material 1. The protocol of this network meta-analysis has been registered on the INPLASY website (https://inplasy.com/inplasy-2020-7-0006), the registration number is INPLASY202070006).

### Selection and Exclusion Criteria

Articles that met the following criteria were included: (1) participants were elderly people (greater than or equal 60 years old) with a diagnosis of MCI; (2) the interventions of the observation group were TCM exercise therapies including Tai Chi, Baduanjin exercise, Qi gong, Liuzijue exercise, finger exercise, etc., and the interventions of the control group were routine nursing intervention or other therapies mentioned above that were different from the intervention group; (3) randomized controlled trials (RCT); (4) at least one outcome indicator used to evaluate the improvement of cognitive function.

Articles with the following characteristics were excluded: (1) studies with unclear descriptions of participants age; (2) studies in which the intervention modality was unclear or combined with medications that could improve cognitive dysfunction, or non-TCM exercise therapies; (3) studies with sample sizes of less than 10 cases per group; (4) studies with incomplete outcome data that could not be extracted; (5) duplicate publications, clinical protocols, case reports, review articles and non-randomized controlled trials.

### Data Extraction and Quality Assessment

Two researchers (HHL and QL) independently reviewed all the articles and extracted the data. The data included information on the characteristics of the author, publication time, country, participants, intervention details, comparison details, duration, and outcome measures. The bias risk assessment tool recommended by the Cochrane Handbook for Systematic Reviews of Interventions 5.1.0 was used to perform quality assessment of included studies (22). The results of data extraction and quality assessment were cross-checked by the two researchers, diversity and disagreement were handled by a third researcher (KQS).

### Outcomes

In this study, we used the Mini-Mental State Examination (MMSE) scale and the Montreal Cognitive Assessment (MoCA) scale as the primary outcomes to assess the cognition function of MCI patients. The MMSE scale is the most commonly used cognitive assessment tool for clinicians, and it has the advantage of simple operation, time saving, and relevance to a wide range of people (23). Compared with the MMSE scale, the MOCA scale adds items that reflect visual spatial function and executive function. It has good sensitivity and specificity in patients with MCI (24). The lower the scores of MMSE and MoCA, the worse the cognitive function. Furthermore, we used the Activity of Daily Living (ADL) scale and the impact on brain function as the secondary outcomes. The ADL scale has 14 entries, which mainly assesses the ability to perform activities of daily living, and consists of the Basic Activities of Daily Living (BADL) scale and the Instrumental Activities of Daily Living (IADL) scale. The BADL scale contains 6 entries assessing the patient's ability to eat, go to the toilet, dress, walk, comb their hair and brush their teeth, and bathe. The IADL scale has 8 entries assessing the patient's ability to make phone calls, shop, do laundry, cook, take medication, and take transportation. A higher ADL score indicates a worse living ability (25). In terms of the impacts on brain function, we conducted a descriptive analysis of the results.

### Statistical Analysis

In this study, the STATA software version 14.0 was utilized to draw network diagrams to visually present the comparison between various therapies, and the Aggregate Data Drug Information System (ADDIS version 1.16.8) and the Bayesian hierarchical model were used for pair-wise meta-analysis and network meta-analysis. The odds ratio (OR) was measured for dichotomous outcomes, and the mean difference (MD) was used for continuous outcomes. The statistics results were presented with an estimated value and 95% confidence interval (CI), and the significance level was set to α = 0.05. If the heterogeneity test results indicate that there was no heterogeneity, the network meta-analysis would be directly performed; otherwise, the analysis and description of the source of the heterogeneity would be implemented. In terms of inconsistency testing, we adopted the node-split model for analysis. When *P* > 0.05, it showed that there was no significant difference between direct and indirect comparison, and the consistency model was used; otherwise, the inconsistency model would be conducted simultaneously. Additionally, we used the potential scale reduction factor (PSRF) to evaluate the convergence of the model. The closer the PSRF value was to 1, the better the convergence of the model (26).

### Assessment of Publication Bias

Funnel plot was generated using the STATA software version 14.0, and the publication bias was evaluated by judging whether the funnel plot was symmetrically distributed. If the distribution was symmetrical, it meant that there was no publication bias.

## Results

### Study Identification and Selection

**Figure 1:**
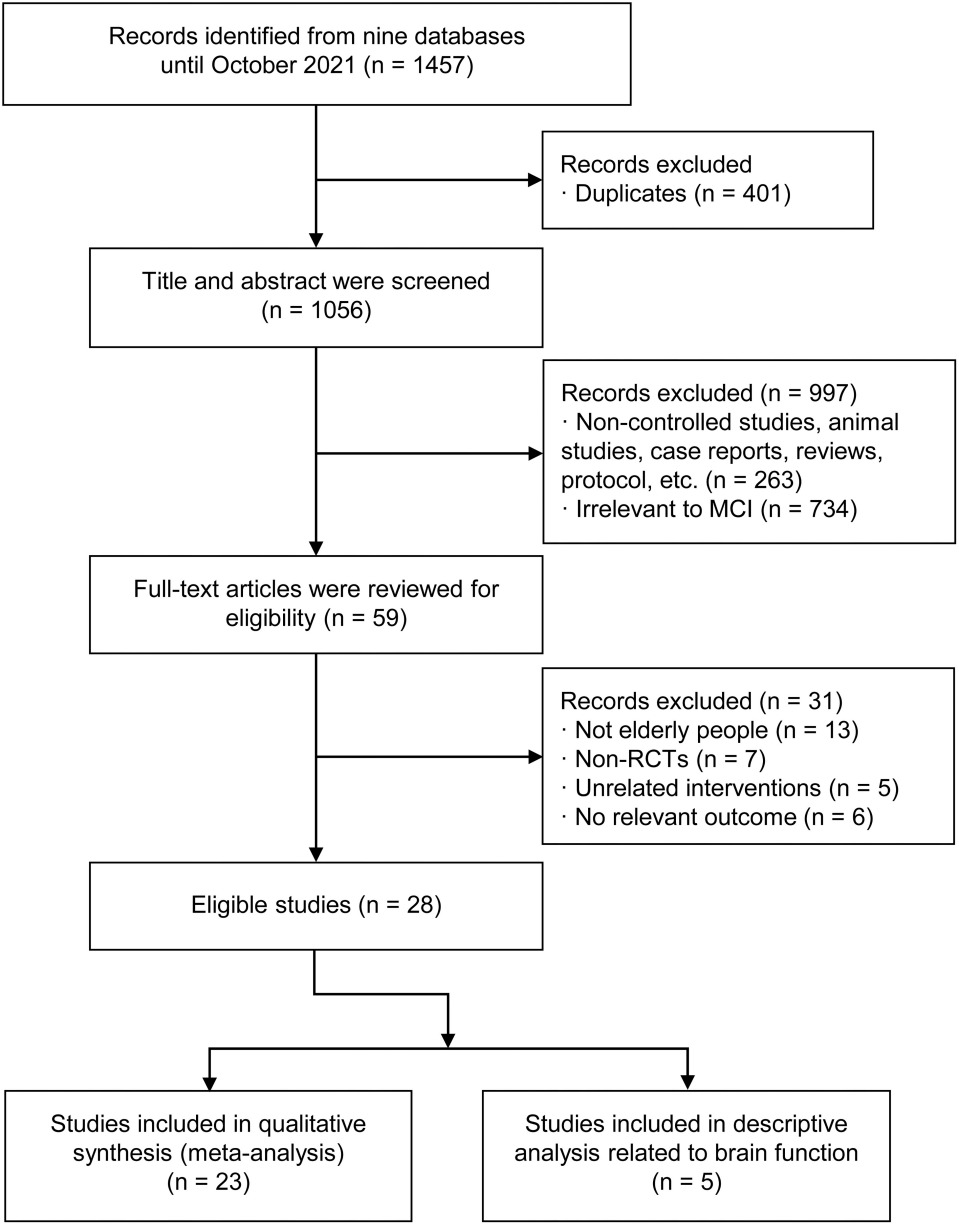
The process of selection of the eligible studies.

Through a systematic search, a total of 1,457 relevant articles were collected from the nine databases. After removing duplications, 1,056 articles remained. Then titles and abstracts were screened by two independent reviewers, and 997 studies including non-controlled studies, animal studies, case reports, reviews, protocols and studies that obviously irrelevant to MCI were excluded. After further reading of the full text of the remaining articles, we excluded 31 studies based on the inclusion criteria, including 13 articles with non-elderly people (60 years or older), 7 non-RCTs, and 11 unrelated intervention or outcome articles. Finally, 23 published RCTs including 2,282 patients were included in this network meta-analysis. These studies were all from China and were published from 2011 to 2021. The baselines for gender, age and sample size were basically the same among these studies. The TCM interventions used in the observation group included Tai Chi, Baduanjin exercise, Luzijue exercise, and finger exercise, while the interventions used in the control group included stretching and toning exercise, health education, routine daily activities and usual care. The process of selection of the eligible studies was shown in Figure 1, and the characteristics of the selected studies in this network meta-analysis were presented in Table 1.

**Table 1:** Characteristics of included studies in this network meta-analysis.

**References**	**Country**	**Diagnosis**	**Participant (O/C)**	**Age (Y)**	**Gender (M/F)**	**Interventions**	**Duration (Week)**	**Outcomes**
Lam et al. (27)	China	aMCI	135/194	>65	Not available	TC group: 24-style Tai Chi, 30 min per day and no less than 3 times per week. STE group: stretching and toning exercise	20	MMSE, ADAS-Cog, CVFT
Geng (28)	China	MCI	35/35	>60	Not available	FE group: finger exercise, per day UC group: usual care	12	MoCA, MMSE, ADL
Qu (29)	China	MCI	43/43	65–89	51/35	FE group: finger exercise, per day UC group: usual care	12	MoCA, MMSE, ADL
Wang et al. (30)	China	MCI with diabetes	30/30	66–90	31/29	FE group: finger exercise, 30 min per day, 5 days per week. UC group: usual care	24	MMSE, ADL
Zheng et al. (31)	China	MCI	45/43	60–78	Not available	LZJE group: Liuzijue exercise, 30 min per day and no less than 5 times per week HE group: health education	24	MoCA, MMSE
Lam et al. (32)	China	MCI	96/169	>65	Not available	TC group: 24-style Tai Chi, 30 min per day and 3 times per week. STE group: stretch and toning exercise	48	MMSE, ADAS-Cog, CVFT
Liu et al. (33)	China	MCI	28/29	69 ± 10.3	Not available	BDJE group: Baduanjin exercise, 60 min per day and 6 times per week. RDA group: routine daily activities	24	MoCA
Zhu et al. (34)	China	MCI with diabetes	37/41	≥60	Not available	BDJE group: Baduanjin exercise, 40 min per day and 3 times per week. RDA group: routine daily activities	24	MoCA, ADL
Chen et al. (35)	China	MCI	33/32	≥60	Not available	FE group: finger exercise, 30 min per day. HE group: health education	12	MMSE
Li (36)	China	MCI	28/29	≥60	15/42	BDJE group: Baduanjin exercise, 60 min per day and 3 times per week. HE group: health education	24	MoCA, WMS, AVLT
Lin (37)	China	MCI	49/49	60–73	41/57	BDJE group: Baduanjin exercise, 30 min per time, 2 times per day. HE group: health education	24	MoCA, MMSE, WMS
Zhang et al. (38)	China	MCI	60/60	65–83	48/72	FE group: finger exercise, 60 min per day RDA group: routine daily activities	24	MoCA, MMSE, ADL
Chen et al. (39)	China	MCI	30/30	≥60	26/34	LZJE group: Liuzijue exercise, 90 min expert guidance per week, and exercise at home the rest of time. RDA group: routine daily activities	12	MMSE, EEG
Lin (40)	China	MCI	47/47	63–78	58/36	BDJE group: Baduanjin exercise, 6 times per week. HE group: health education	24	MoCA, WMS, MMSE, ADL
Xia (41)	China	MCI	31/31	≥60	Not available	BDJE group: Baduanjin exercise, 60 min per time, 3 times per week. HE group: health education	24	MoCA
Siu and Lee (42)	China	MCI	80/80	≥60	42/118	TC group: 24-style Tai Chi, 60 min per time and 2 times per week. UC group: usual care	16	MMSE
Liu et al. (43)	China	MCI	30/30	≥60	38/22	BDJE group: Baduanjin exercise, 60min per time, 6 times per week. HE group: health education	24	MoCA
Zhao and Tao (44)	China	MCI	27/28	≥60	21/34	FE group: finger exercise, 15min per time, 3 times per day UC group: usual care	12	MoCA, ADL
Xue (45)	China	MCI	35/34	>60	29/40	FE group: finger exercise, 10min per time, 3 times per day HE group: health education	24	MMSE, MoCA, ADL
Ye (46)	China	MCI	20/20	>60	Not available	BDJE group: Baduanjin exercise, 60 min per time, 3 times per week. HE group: health education	24	MoCA, WMS
Li et al. (47)	China	MCI	70/70	≥60	67/73	FE group: finger exercise, 30 min per time, 3 times per day UC group: usual care	12	MMSE
Xia (48)	China	MCI	51/51	Mean age = 67.68	39/63	BDJE group: Baduanjin exercise, 60 min per day and 3 times per week. HE group: health education	24	MoCA, Trail Making Test
Sun et al. (49)	China	MCI	29/28	65–68	21/36	BDJE group: Baduanjin exercise, 50min per time, 3 times per week. HE group: health education	24	MoCA, RBMT-II, DSST, SPPB

*O, observation group; C, control group; Y, year; M, male; F, female; aMCI, amnestic-type mild cognitive impairment; MCI, mild cognitive impairment; CDR, Clinical Dementia Rating; MMSE, mini-mental state examination; ADAS-Cog, Alzheimer's Disease Assessment Scale-Cognitive subscale; CVFT, category verbal fluency tests; MoCA, Montreal Cognitive Assessment; ADL, activity of daily living; WMS, Wechsler Memory Scale; AVLT, Auditory Verbal Learning Test; EEG, electroencephalo-graph; RBMT-II, Rivermead behavioral memory test second edition; DSST, digit symbol substitution test; SPPB, short physical performance battery*.

### Study Quality

**Figure 2:**
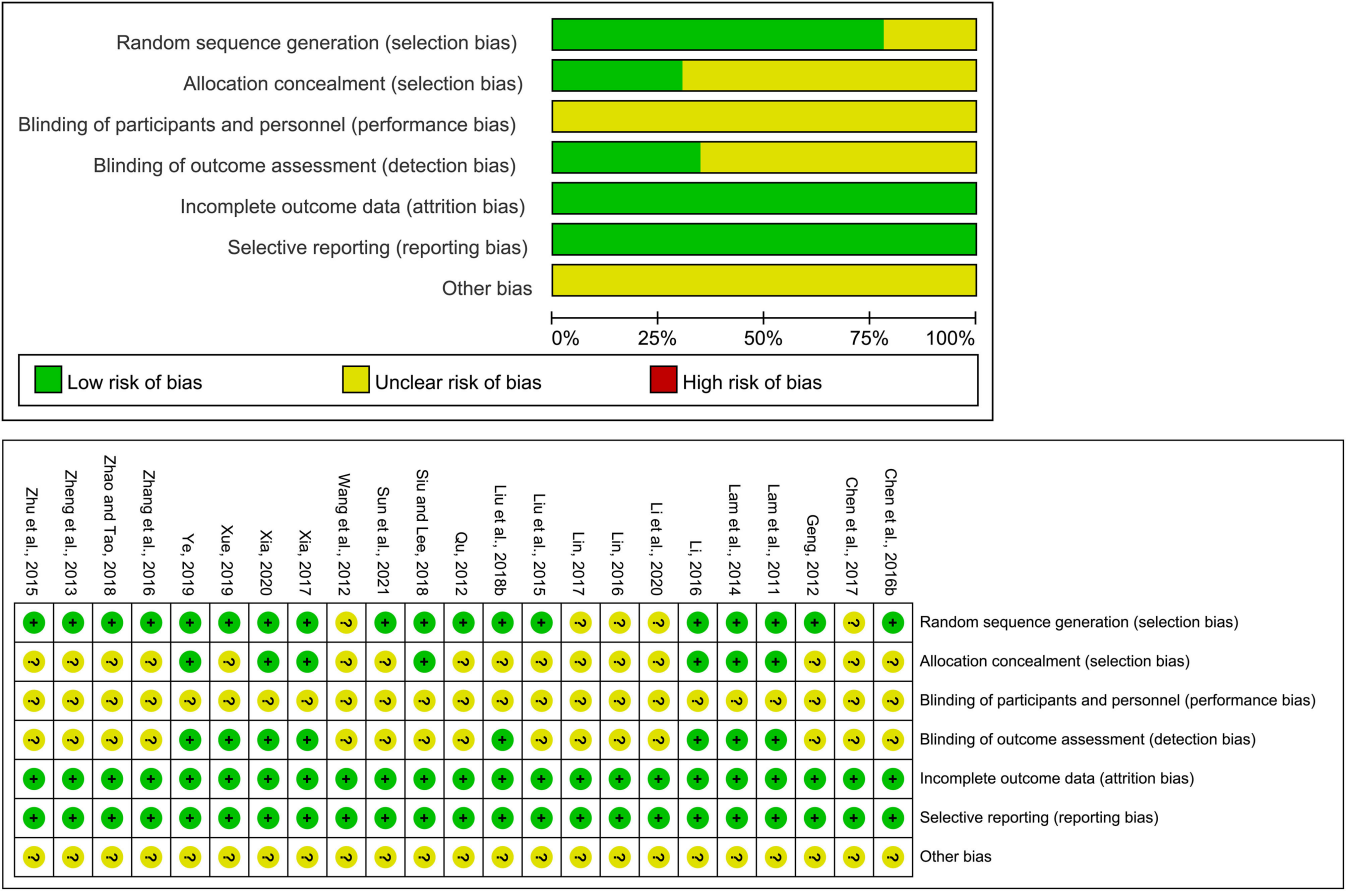
Quality assessment of selected studies.

We assessed the risk of bias for eligible studies using the Cochrane Risk of Bias tool. Regarding the generation of randomization sequence, 18 studies used an appropriate random generation method, such as a random sequence generation process using a computerized random number generator or a random number table, and 5 studies did not mention the randomization methods. Seven studies reported allocation concealment method and none described the use of blinding of researchers and participants, which may be explained by the fact that exercise therapy as a non-drug therapy cannot be ignored by subjects and operators. Eight studies mentioned blinding of outcome assessment. In all studies, the baseline characteristics of the groups were comparable and all the studies reported complete data. Figure 2 shows the summary risk of bias for selected studies.

### Pair-Wise Meta-Analysis

After synthesizing studies with the same interventions, we performed a classic pair-wise meta-analysis using a random effects model to compare the differences between TCM exercise therapies and non-TCM exercise therapies, and the definition of non-TCM exercise therapies includes stretching and toning exercise, usual care, health education and routine daily activities. As for the MMSE outcome, Baduanjin exercise (MD: 3.19; 95% CI: 2.12, 4.27), Tai Chi (MD: 1.30; 95% CI: 0.52, 2.07), Liuzijue exercise (MD: 0.55; 95% CI: −0.17, 1.28), finger exercise (MD: 1.53; 95% CI: 0.62, 2.44) were more efficient than non-TCM exercise therapies. For the MoCA, Baduanjin exercise (MD: 3.27; 95% CI: 2.08, 4.46), Liuzijue exercise (MD: 2.35; 95% CI: 0.26, 4.44), finger exercise (MD: 2.17; 95% CI: 1.52, 2.83) were statistically more effective than non-TCM exercise therapies. For the ADL outcome, Baduanjin exercise (MD: −1.75; 95% CI: −2.26, −1.24), finger exercise (MD: −2.13; 95% CI: −3.30, −0.96) were better than non-TCM exercise therapies. The results of the pair-wise meta-analysis are shown in Supplementary Material 2.

### Network Meta-Analysis

#### Primary Outcome: MMSE and MoCA

**Figure 3:**
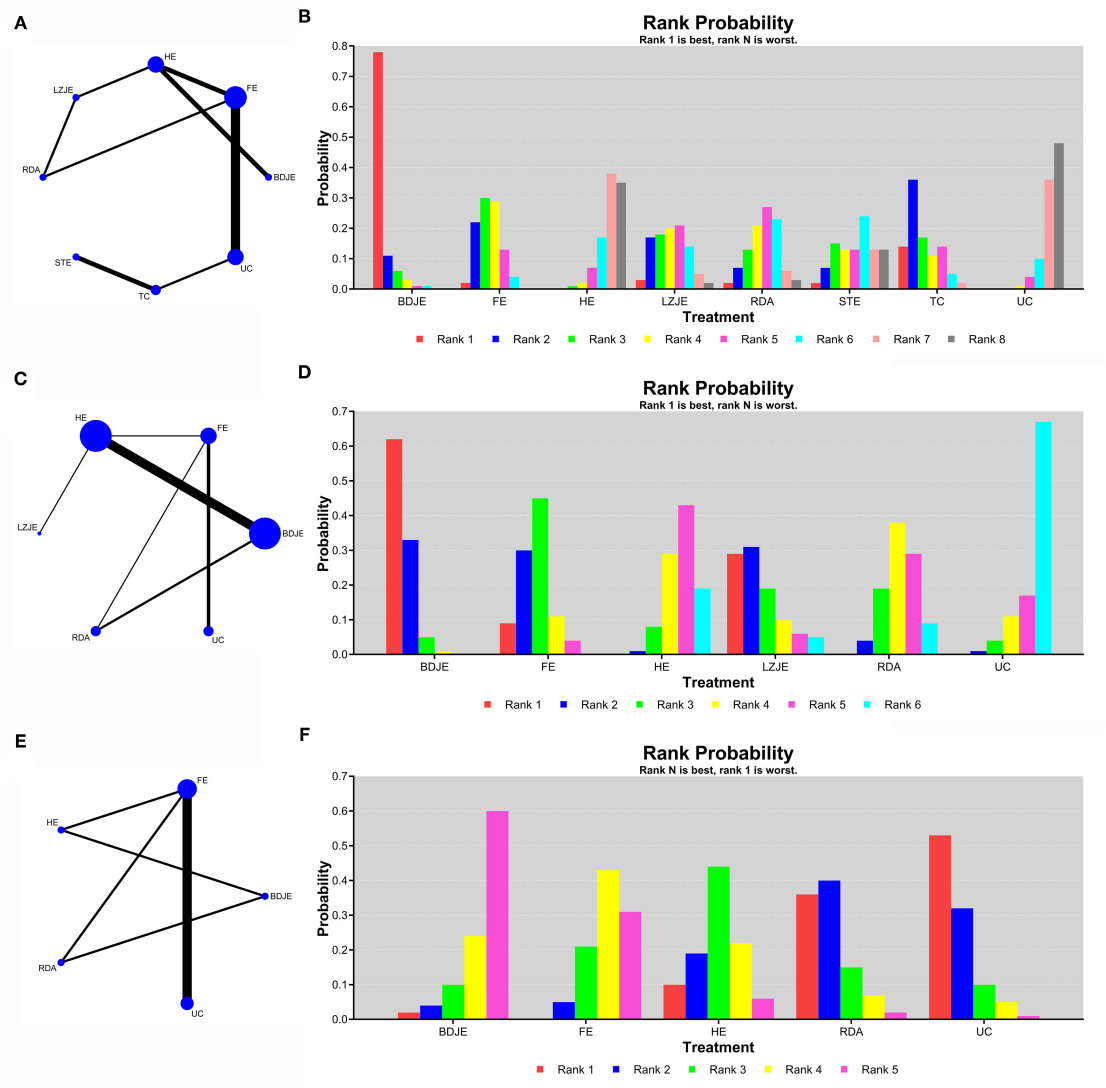
The network comparisons for the outcome of MMSE **(A)**, MoCA **(C)** and ADL **(E)**. The ranking probability of MMSE **(B)**, MoCA **(D)** and ADL **(F)**. BDJE, Baduanjin exercise; FE, finger exercise; HE, health education; LZJE, Liuzijue exercise; RDA, routine daily activities; STE, stretching and toning exercise; TC, Tai Chi; UC, usual care.

We performed a network meta-analysis to compare the effects of different interventions for MCI, and we also ranked the comparison results. As shown in Figures 3A,C, the network diagrams with MMSE and MoCA as the outcome indicators contained 14 studies and 8 intervention methods (MMSE) and 16 studies and 6 intervention methods (MoCA). The results of node-splitting analysis indicated that the *p* values were > 0.05, and the values of PSRF were equal to 1, which indicated that the model was convergent and the results were relatively stable. Therefore, we chose the consistency model in the subsequent network analysis.

The results of network meta-analysis for the MMSE are shown in Table 2. Compared with usual care, Baduanjin exercise (MD: 3.38; 95% CI: 0.97, 5.82), finger exercise (MD: 1.80; 95% CI: 0.72, 2.89), health education (MD: 0.17; 95% CI: −1.53, 1.86), Liuzijue exercise (MD: 1.54; 95% CI: −0.64, 3.65), routine daily activities (MD: 1.31; 95% CI: −0.67, 3.29), stretching and toning exercise (MD: 1.09; 95% CI: −1.43, 3.60), and Tai Chi (MD: 2.04; 95% CI: −0.02, 4.13) all had better curative effects. As shown in the probability ranking chart of Figure 3B, the Baduanjin exercise had the highest probability (78%) of becoming the best intervention for MCI in the elderly, followed by Tai Chi (36%).

**Table 2:** The consistency model of MMSE.

BDJE	−1.60 (−3.78, 0.56)	−3.23 (−4.94, −1.53)	−1.88 (−4.40, 0.63)	−2.10 (−4.59, 0.44)	−2.28 (−5.79, 1.20)	−1.33 (−4.54, 1.76)	−3.38 (−5.82, −0.97)
1.60 (−0.56, 3.78)	FE	−1.62 (−2.92, −0.31)	−0.26 (−2.17, 1.58)	−0.49 (−2.14, 1.16)	−0.68 (−3.48, 2.08)	0.26 (−2.05, 2.59)	−1.80 (−2.89, −0.72)
3.23 (1.53, 4.94)	1.62 (0.31, 2.92)	HE	1.37 (−0.43, 3.12)	1.13 (−0.67, 2.98)	0.93 (−2.12, 3.97)	1.88 (−0.81, 4.52)	−0.17 (−1.86, 1.53)
1.88 (−0.63, 4.40)	0.26 (−1.58, 2.17)	−1.37 (−3.12, 0.43)	LZJE	−0.23 (−1.90, 1.41)	−0.42 (−3.84, 2.94)	0.52 (−2.40, 3.42)	−1.54 (−3.65, 0.64)
2.10 (−0.44, 4.59)	0.49 (−1.16, 2.14)	−1.13 (−2.98, 0.67)	0.23 (−1.41, 1.90)	RDA	−0.20 (−3.38, 3.03)	0.76 (−2.03, 3.55)	−1.31 (−3.29, 0.67)
2.28 (−1.20, 5.79)	0.68 (−2.08, 3.48)	−0.93 (−3.97, 2.12)	0.42 (−2.94, 3.84)	0.20 (−3.03, 3.38)	STE	0.95 (−0.51, 2.43)	−1.09 (−3.60, 1.43)
1.33 (−1.76, 4.54)	−0.26 (−2.59, 2.05)	−1.88 (−4.52, 0.81)	−0.52 (−3.42, 2.40)	−0.76 (−3.55, 2.03)	−0.95 (−2.43, 0.51)	TC	−2.04 (−4.13, 0.02)
3.38 (0.97, 5.82)	1.80 (0.72, 2.89)	0.17 (−1.53, 1.86)	1.54 (−0.64, 3.65)	1.31 (−0.67, 3.29)	1.09 (−1.43, 3.60)	2.04 (−0.02, 4.13)	UC

*BDJE, Baduanjin exercise; FE, finger exercise; HE, health education; LZJE, Liuzijue exercise; RDA, routine daily activities; STE, stretching and toning exercise; TC, Tai Chi; UC, usual care*.

The results of network meta-analysis for the MoCA are shown in Table 3. Compared with usual care, Baduanjin exercise (MD: 4.36; 95% CI: 0.89, 7.77), finger exercise (MD: 2.81; 95% CI: 0.79, 4.89), health education (MD: 1.01; 95% CI: −2.37, 4.41), Liuzijue exercise (MD: 3.34; 95% CI: −1.72, 8.57), routine daily activities (MD: 1.44; 95% CI: −1.96, 4.84) all had better curative effects. As shown in Figure 3D, the Baduanjin exercise had the highest probability (62%) of becoming the best intervention for MCI in the elderly.

**Table 3:** The consistency model of MoCA.

BDJE	−1.55 (−4.37, 1.16)	−3.35 (−4.58, −2.15)	−1.02 (−5.01, 3.08)	−2.93 (−5.28, −0.50)	−4.36 (−7.77, −0.89)
1.55 (−1.16, 4.37)	FE	−1.79 (−4.50, 0.97)	0.53 (−4.18, 5.33)	−1.36 (−4.03, 1.39)	−2.81 (−4.89, −0.79)
3.35 (2.15, 4.58)	1.79 (−0.97, 4.50)	HE	2.34 (−1.47, 6.27)	0.42 (−2.06, 3.04)	−1.01 (−4.41, 2.37)
1.02 (−3.08, 5.01)	−0.53 (−5.33, 4.18)	−2.34 (−6.27, 1.47)	LZJE	−1.94 (−6.46, 2.73)	−3.34 (−8.57, 1.72)
2.93 (0.50, 5.28)	1.36 (−1.39, 4.03)	−0.42 (−3.04, 2.06)	1.94 (−2.73, 6.46)	RDA	−1.44 (−4.84, 1.96)
4.36 (0.89, 7.77)	2.81 (0.79, 4.89)	1.01 (−2.37, 4.41)	3.34 (−1.72, 8.57)	1.44 (−1.96, 4.84)	UC

*BDJE, Baduanjin exercise; FE, finger exercise; HE, health education; LZJE, Liuzijue exercise; RDA, routine daily activities; UC, usual care*.

#### Secondary Outcome: ADL

As shown in Figure 3E, the network diagram with ADL as the outcome indicator contained 8 studies and 5 intervention methods. The consistency model was adopted in order to compare these different interventions after node-splitting analysis. The results in Table 4 showed that comparing with usual care, the Baduanjin exercise (MD: −3.11; 95% CI: −7.82, 1.69), finger exercise (MD: −2.89; 95% CI: −4.55, −0.31), health education (MD: −1.56; 95% CI: −5.66, 2.74), and routine daily activities (MD: −0.35; 95% CI: −4.57, 3.82) had better curative effects. Similarly, Figure 3F showed the probabilistic ranking of ADL, the results suggested that the ranking of Baduanjin exercise was the best (60%), it might be the most effective intervention to improve the ADL score of elderly MCI patients, followed by finger exercise (43%).

**Table 4:** The consistency model of ADL.

BDJE	0.63 (−3.48, 4.75)	1.58 (−1.97, 5.03)	2.74 (−1.08, 6.50)	3.11 (−1.69, 7.82)
−0.63 (−4.75, 3.48)	FE	0.94 (−2.66, 4.56)	2.15 (−1.56, 5.64)	2.49 (0.31, 4.55)
−1.58 (−5.03, 1.97)	−0.94 (−4.56, 2.66)	HE	1.17 (−3.18, 5.31)	1.56 (−2.74, 5.66)
−2.74 (−6.50, 1.08)	−2.15 (−5.64, 1.56)	−1.17 (−5.31, 3.18)	RDA	0.35 (−3.82, 4.57)
−3.11 (−7.82, 1.69)	−2.49 (−4.55, −0.31)	−1.56 (−5.66, 2.74)	−0.35 (−4.57, 3.82)	UC

*BDJE, Baduanjin exercise; FE, finger exercise; HE, health education; RDA, routine daily activities; UC, usual care*.

### The Impacts on Brain Function

There were 5 studies have observed the impacts of TCM exercise therapies (Tai Chi and Baduanjin exercise) on the brain function of elderly patients with MCI. Tao et al. found that Tai Chi and Baduanjin exercise (1 h per time, 3 times per week, for 24 weeks) can significantly improve the memory ability of elderly patients with MCI, and the curative effect were better than that of the health education group. The functional magnetic resonance imaging of brain showed that Tai Chi can reduce the resting state functional connection between the bilateral dorsolateral prefrontal cortex and the anterior cingulate cortex and the left superior (50), and increase fractional amplitude of low-frequency fluctuations in the dorsolateral prefrontal cortex (51); while Baduanjin exercise can reduce the resting state functional connection between the bilateral dorsolateral prefrontal cortex and the left putamen and insula (50), and increase fractional amplitude of low-frequency fluctuations in medial prefrontal cortex (51). In 2019, Tao et al. also found that Baduanjin exercise (1 h per time, 3 times per week, for 24 weeks) can increase the gray matter volume of the right hippocampus and bilateral anterior cingulate gyrus, and increase the resting state functional connection between the hippocampus and the right angular gyrus (52). Additionally, further studies found that Baduanjin exercise (1 hour per time, 3 times per week, for 24 weeks) can improve the selective attention of elderly patients with MCI, reduce the functional connectivity of the dorsal attention network, and modulate the brain's intrinsic functional connectivity and the norepinephrine and dopamine systems (19, 53).

### Sensitivity Analysis

Sensitivity analysis were performed to assess the reliability of the results by excluding studies with less than 12 weeks of duration, sample sizes of less than 60 cases, and studies with a high risk of bias. The results showed significant heterogeneity in the pair-wise meta-analysis of MMSE outcome for finger exercise, which was eliminated when the study was excluded (47), considered to be related to the lower methodological quality of the study. The remaining results were consistent and the conclusions were reliable.

### Publication Bias

**Figure 4:**
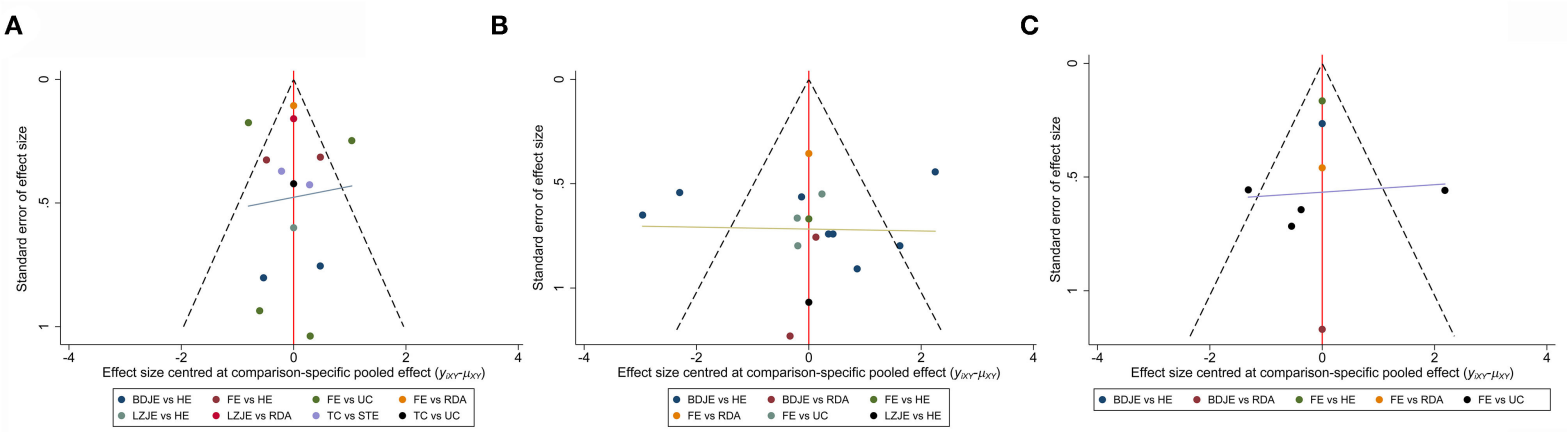
The funnel plots of MMSE **(A)**, MoCA **(B)** and ADL **(C)**.

We used MMSE, MoCA and ADL as the outcome indicators to generate funnel plots through STATA software version 14.0. The results suggested that the funnel plots in MMSE (Figure 4A) and MoCA (Figure 4B) were basically stacked, and it can be considered that there was no publication bias. However, the ADL funnel plot had poor symmetry and may have publication bias, which may be linked to the small number of included studies and the small total sample size (Figure 4C).

## Discussion

As a global issue of aging, cognitive impairment severely affects the daily life of patients. The complex pathogenesis of cognitive impairment also limits the development of interventions with specific efficacy and safety. Exercise therapy, an alternative therapy to improve general or local function through active or passive activities, has been widely promoted in recent years. In 2017, the practice guidelines for MCI published by the American Academy of Neurology recommended exercise twice a week for patients with MCI (10). In China, the “Healthy China 2030” plan promulgated by the Central Committee of the Communist Party of China also clearly stated that the role of TCM in the treatment of chronic diseases should be brought into full play (54).

According to the classification of cognitive domains, mild cognitive impairment can be divided into amnestic MCI, where there is impairment of memory function, and non-amnestic MCI, where there is impairment of other cognitive domains. Meanwhile, MCI can be caused by different diseases, such as Alzheimer's disease, cerebral small vessel disease, Lewy body disease, frontotemporal lobar degeneration, diabetes mellitus, etc. (2). One of the studies included in this study investigated the effect of Tai Chi on patients with amnestic MCI (27). Two studies investigated the effects of finger exercise (30) and Baduanjin exercise (34) on patients with diabetes-related MCI, respectively, while the remaining studies did not report on the typology and etiology of MCI. It is worth mentioning that the onset of diabetes mellitus is more insidious in MCI patients, and the cognitive impairment may lead to patients missing medications or forgetting to eat after insulin injection, etc., thus aggravating glucose metabolism disorders (34). Available evidence confirms that exercise therapy is one of the effective adjuvant therapies for diabetes, which can effectively improve patients' blood glucose levels, reduce target organ and microangiopathy, and improve cognitive function (55, 56).

In this study, we conducted a network meta-analysis of potential TCM exercise therapies for MCI in the elderly, and used Bayesian statistics to rank the curative effects of different therapies on different outcomes, and obtained the recommended therapy with the best curative effect. We found that the four TCM exercise therapies (Baduanjin exercise, Tai Chi, Liuzijue exercise and finger exercise) are superior to other non-TCM exercise therapies (health education, routine daily activities, stretching and toning exercise and usual care). Furthermore, among the four intervention methods, Baduanjin exercise has the best effect on the improvement of MMSE, MoCA and ADL in elderly patients with MCI.

Baduanjin is an exercise method composed of eight groups of movements, which has the advantages of being easy to learn and moderate exercise, and helps to improve blood circulation in the neck and head, improve cardiopulmonary function, and stimulate the cerebral cortex and sympathetic nerve excitation, and is commonly used for some chronic diseases of the elderly such as hypertension, insomnia, menopausal syndrome and dementia (57). Several studies have found that Baduanjin exercise can improve the attention, executive ability and memory of patients with cognitive impairment (19, 53, 58, 59).

As a traditional method of health care, Tai Chi is a moderate intensity, safe and reliable aerobic fitness exercise with the effect of harmonizing Qi and blood, enhancing heart function and improving balance, and is commonly used in chronic cardiovascular disease, chronic lung disease and degenerative joint disease. It generally includes 12 or 24 postures, and each exercise lasts 30–60 min. Several studies have shown that Tai Chi can improve the cognitive function of elderly people in the community and improve the quality of life (60–62). However, in this study, we found that Tai Chi ranked second in terms of probability of improving MMSE scores in MCI patients, after Baduanjin exercise, which may be related to the fact that Tai Chi has a large number of stances and complex forms, and therefore it may not be the most suitable practice for elderly patients with cognitive impairment, especially dementia. However, more in-depth comparative studies are needed to verify this.

Liuzijue exercise is a kind of exhalation therapy which involves the practice of pronouncing six different Chinese characters to improve the flow of Qi and blood in the meridians of different organs. It has the effect of strengthening tissue function and improving respiratory function, and is mostly used in the treatment of chronic lung diseases (63, 64). However, evidence from our research so far has found that the Liuzijue exercise may be less suitable for patients with cognitive impairment to practice. However, again, this needs further confirmation.

Notably, our study found that finger exercise had the second highest probability ranking in term of improving ADL and may be a potential exercise therapy to improve ADL in MCI patients. Finger exercise is a therapy that stimulates acupuncture points and meridians of the hand by moving, tapping, pressing, etc. (17). The meridian theory and the holographic theory of TCM believe that the human hands are covered with reflective areas of various organs. Stimulating the acupoints and meridians of the hands can promote the blood circulation of the corresponding organs, so as to achieve the effect of life cultivation and health preservation. Therefore, finger exercise is mostly applied to eliminate fatigue, reduce mental burden and relieve tension (65). Previous studies have concluded that the structure and function of the brain are highly plastic (66), and fine hand exercises can activate the functions of the cerebral cortex in multiple brain areas, thereby delaying the decline of cognitive function (67, 68). In addition, the training of fine hand exercises can intuitively improve the patient's self-care ability in daily life, such as eating, washing face, brushing teeth, dressing, bathing and other actions that highly dependent on hand functions (69, 70). Therefore, finger exercise may be a potential therapy that can improve MCI, which may be linked to improving the function of patients' hands and improving their self-care ability. This still needs further validation.

In addition, in this study, we also briefly reviewed studies related to the impacts of TCM exercise therapies on brain function. In recent years, the development of functional magnetic resonance imaging (fMRI) has aided the study of cognitive impairment. Combining neuropsychological measurements and fMRI can help to understand changes in brain function during changes in different cognitive domains, which not only provides an objective basis for patients' cognitive impairment, but also provides a visual method to explore the mechanisms of cognitive impairment. Some studies have confirmed that the occurrence of MCI may be associated with reduced perfusion and metabolism in the hippocampus, temporoparietal and posterior cingulate gyrus, or atrophy of the hippocampus and internal olfactory cortex (71–73). In parallel, recent studies have also found that functional connectivity of brain structures and functions such as the default mode network, central executive network and salience network are also altered in patients with cognitive impairment (48, 74, 75). This systematic review summarized the effects of TCM exercise therapies on brain function in patients with MCI and found that Tai Chi and Baduanjin exercise can regulate the resting state functional connection and fractional amplitude of low-frequency fluctuations, and increase the gray matter volume of the hippocampus and the anterior cingulate cortex. These above findings indicated that the mechanism of TCM exercise therapies in improving MCI may be related to the regulation of cognitive-related brain function and structure. However, given the paucity of relevant studies, further exploration is still needed.

Several limitations exist in our study. First, all included studies were from China, the lack of other ethnic studies may lead to some bias and less convincing results. Second, although there was no obvious inconsistency or heterogeneity shown in this network meta-analysis, the small sample size, fewer included studies, and the diversity of treatment methods in the control group may lead to a certain potential bias, which may overestimate the effectiveness of TCM exercise therapies, thereby affecting the reliability of the results. Third, due to the characteristics of TCM exercise therapies, it is not possible or difficult to blind the participants during implementation, thus leads to the existence of bias. Therefore, large-scale RCTs using strict methodology are necessary in order to examine our findings. Nevertheless, our study still provides reliable information for the treatment decisions of elderly patients with MCI.

## Conclusion

The results of this study provide some evidence that TCM exercise therapies may improve the cognitive function of elderly patients with MCI. Among the four therapies included, the Baduanjin exercise may be the preferred therapy for treatment of MCI in the elderly, and the mechanism of Baduanjin exercise in improving MCI may be related to the regulation of cognitive-related brain function and structure.

## Data Availability Statement

The original contributions presented in the study are included in the article/Supplementary Material, further inquiries can be directed to the corresponding author/s.

## Author Contributions

KS and JY designed this study. KS, JY, and ML searched the literature. HL and QL collected the data. KS and SL performed all analysis. KS, JY, and SL wrote the original draft of the manuscript. All authors contributed to writing of the manuscript.

## Funding

This work was funded by grants from the National Natural Science Foundation of China (Nos. U2004131 and 82174473) and Henan Administration of Traditional Chinese Medicine (2018JDZX011).

## Conflict of Interest

The authors declare that the research was conducted in the absence of any commercial or financial relationships that could be construed as a potential conflict of interest.

## Publisher's Note

All claims expressed in this article are solely those of the authors and do not necessarily represent those of their affiliated organizations, or those of the publisher, the editors and the reviewers. Any product that may be evaluated in this article, or claim that may be made by its manufacturer, is not guaranteed or endorsed by the publisher.
