# Supplementary material for: The Comparative Effectiveness of Traditional Chinese Medicine Exercise Therapies in Elderly People With Mild Cognitive Impairment: A Systematic Review and Network Meta-Analysis
Source: Front Neurol. 2022 Mar 16;13:775190. doi: 10.3389/fneur.2022.775190 (PMC8966650; doi:10.3389/fneur.2022.775190)
Supplement: Supplementary file 1 [file Table_1.DOCX]

**Supplementary file 1. The search strategy**

| **Number** | **Database** |  | **Search terms** |
| --- | --- | --- | --- |
| 1 | China National Knowledge Infrastructure  (CNKI) | #1 | (主题=运动疗法 + 运动治疗 + 传统运动 + 传统运动疗法 + 太极拳 + 八段锦 + 六字诀 + 手指操 + 身心运动 + 气功 + 五禽戏 + 易筋经) AND (主题=轻度认知障碍 + 轻度认知功能障碍 + 轻度认知功能损害 + 轻度认知功能损伤 + 轻度认知损害 + 轻度认知损伤 + 轻微认知功能损害) AND (全文=随机) |
| 2 | Wangfang | #1 | 主题:(运动疗法 or 运动治疗 or 传统运动 or 传统运动疗法 or 太极拳 or 八段锦 or 六字诀 or 手指操 or 身心运动 or 气功 or 五禽戏 or 易筋经) and 主题:(轻度认知障碍 or 轻度认知功能障碍 or 轻度认知功能损害 or 轻度认知功能损伤 or 轻度认知损害 or 轻度认知损伤 or 轻微认知功能损害) and 全部:(随机) |
| 3 | Chinese Biomedical Medicine  (CBM) | #1 | ( "运动疗法"[常用字段] OR "运动治疗"[常用字段] OR "传统运动"[常用字段] OR "传统运动疗法"[常用字段] OR "太极拳"[常用字段] OR "八段锦"[常用字段] OR "六字诀"[常用字段] OR "手指操"[常用字段] OR "身心运动"[常用字段] OR "气功"[常用字段] OR "五禽戏"[常用字段] OR "易筋经"[常用字段]) AND ( "轻度认知障碍"[常用字段] OR "轻度认知功能障碍"[常用字段] OR "轻度认知功能损害"[常用字段] OR "轻度认知功能损伤"[常用字段] OR "轻度认知损害"[常用字段] OR "轻度认知损伤"[常用字段] OR "轻微认知功能损害"[常用字段]) AND "随机"[常用字段:智能] |
| 4 | China Science and Technology Journal Database  (CQVIP) | #1 | ((((((((((题名或关键词="运动疗法" OR 题名或关键词="运动治疗") OR 题名或关键词="传统运动") OR 题名或关键词="传统运动疗法") OR 题名或关键词="太极拳") OR 题名或关键词="八段锦") OR 题名或关键词="六字诀") OR 题名或关键词="手指操") OR 题名或关键词="身心运动") OR 题名或关键词="气功") OR 题名或关键词="五禽戏") OR 题名或关键词="易筋经") AND ((((((题名或关键词="轻度认知障碍" OR 题名或关键词="轻度认知功能障碍") OR 题名或关键词="轻度认知功能损害") OR 题名或关键词="轻度认知功能损伤") OR 题名或关键词="轻度认知损害") OR 题名或关键词="轻度认知损伤") OR 题名或关键词="轻微认知功能损害")) |
| 5 | Pubmed | #1 | ((exercise therapy[Title/Abstract] OR exercise therapies[Title/Abstract] OR remedial exercise[Title/Abstract] OR rehabilitation exercise[Title/Abstract] OR traditional Chinese medicine exercise therapy[Title/Abstract] OR mind-body[Title/Abstract] OR Tai Ji[Title/Abstract] OR Tai-ji[Title/Abstract] OR Tai Chi[Title/Abstract] OR Tai Ji Quan[Title/Abstract] OR Baduanjin[Title/Abstract] OR Baduan jin[Title/Abstract] OR Qi gong[Title/Abstract] OR Liuzijue[Title/Abstract] OR Liuzi jue[Title/Abstract] OR finger exercise[Title/Abstract] OR finger gym[Title/Abstract] OR Wuqin xi[Title/Abstract] OR Wuqinxi[Title/Abstract] OR five animal exercise[Title/Abstract] OR Yijin jing[Title/Abstract] OR Yijinjing[Title/Abstract] OR classics of tendon changing[Title/Abstract]) AND (mild cognitive impairment[Title/Abstract] OR cognitive impairment[Title/Abstract] OR cognitive dysfunction[Title/Abstract] OR mild neurocognitive disorder[Title/Abstract] OR cognitive decline[Title/Abstract] OR mental deterioration[Title/Abstract])) AND (randomized controlled trials OR random OR randomly OR allocation OR random allocation OR placebo OR single blind OR double blind OR clinical trials OR randomized control trial OR RCT OR controlled clinical trials) |
| 6 | Web of Science | #1 | (AB=(exercise therapy) OR AB=(exercise therapies) OR AB=(remedial exercise) OR AB=(rehabilitation exercise) OR AB=(traditional Chinese medicine exercise therapy) OR AB=(mind-body) OR AB=(Tai Ji) OR AB=(Tai-ji) OR AB=(Tai Chi) OR AB=(Tai Ji Quan) OR AB=(Baduanjin) OR AB=(Baduan jin) OR AB=(Qi gong) OR AB=(Liuzijue) OR AB=(Liuzi jue) OR AB=(finger exercise) OR AB=(finger gym) OR AB=(Wuqinxi) OR AB=(Wuqin xi) OR AB=(five animal exercise) OR AB=(Yijin jing) OR AB=(Yijinjing) OR AB=(classics of tendon changing)) |
|  |  | #2 | (AB=(mild cognitive impairment) OR AB=(cognitive impairment) OR AB=(cognitive dysfunction) OR AB=(mild neurocognitive disorder) OR AB=(cognitive decline) OR AB=(mental deterioration)) |
|  |  | #3 | (ALL=(randomized controlled trials) OR ALL=(random) OR ALL=(randomly) OR ALL=(allocation) OR ALL=(random allocation) OR ALL=(placebo) OR ALL=(single blind) OR ALL=(double blind) OR ALL=(clinical trials) OR ALL=(randomized control trial) OR ALL=(RCT) OR ALL=(controlled clinical trials)) |
|  |  | #4 | #1 AND #2 AND #3 |
| 7 | EMBASE | #1 | ('exercise therapy':ab,ti OR 'exercise therapies':ab,ti OR 'remedial exercise':ab,ti OR 'rehabilitation exercise':ab,ti OR 'traditional chinese medicine exercise therapy':ab,ti OR 'mind body':ab,ti OR 'tai ji':ab,ti OR 'tai chi':ab,ti OR 'tai ji quan':ab,ti OR baduanjin:ab,ti OR 'baduan jin':ab,ti OR 'qi gong':ab,ti OR liuzijue:ab,ti OR 'liuzi jue':ab,ti OR 'finger exercise':ab,ti OR 'finger gym':ab,ti OR Wuqinxi:ab,ti OR 'Wuqin Xi':ab,ti OR 'five animal exercise':ab,ti OR 'Yijinjing':ab,ti OR 'Yijin Jing':ab,ti OR 'classics of tendon changing':ab,ti) AND ('mild cognitive impairment':ab,ti OR 'cognitive impairment':ab,ti OR 'cognitive dysfunction':ab,ti OR 'mild neurocognitive disorder':ab,ti OR 'cognitive decline':ab,ti OR 'mental deterioration':ab,ti) AND ('randomized controlled trials' OR 'randomly' OR 'allocation' OR 'random allocation' OR 'placebo' OR 'single blind' OR 'double blind' OR 'clinical trials' OR 'randomized control trial' OR 'rct' OR 'controlled clinical trials') |
| 8 | Cochrane Library | #1 | ((exercise therapy*) or (exercise therapies*) or (remedial exercise*) or (rehabilitation exercise*) or (traditional Chinese medicine exercise therapy*) or (mind-body*) or (Tai Ji*) or (Tai-ji*) or (Tai Chi*) or (Tai Ji Quan*) or (baduanjin*) or (baduan jin*) or (Qi gong*) or (liuzijue*) or (liuzi jue*) or (finger exercise*) or (finger gym*)) or (Wuqinxi*) or (Wuqin xi*) or (five animal exercise*) or (Yijinjing*) or (Yijin jing*) or (classics of tendon changing*):ti,ab |
|  |  | #2 | ((cognitive impairment*) or (cognitive dysfunction*) or (mild neurocognitive disorder*) or (cognitive decline*) or (mental deterioration*)):ti,ab |
|  |  | #3 | ((randomv) or (randomlyv) or (allocationv) or (random allocationv) or (placebo*) or (single blindv) or (double blindv) or (clinical trialsv) or (randomized control trialv) or (RCT*) or (controlled clinical trials*)):ti,ab |
|  |  | #4 | #1 AND #2 AND #3 |
