# Supplementary material for: The Comparative Effectiveness of Traditional Chinese Medicine Exercise Therapies in Elderly People With Mild Cognitive Impairment: A Systematic Review and Network Meta-Analysis
Source: Front Neurol. 2022 Mar 16;13:775190. doi: 10.3389/fneur.2022.775190 (PMC8966650; doi:10.3389/fneur.2022.775190)
Supplement: Supplementary file 2 [file Table_2.DOCX]

Supplementary file 2

MMSE


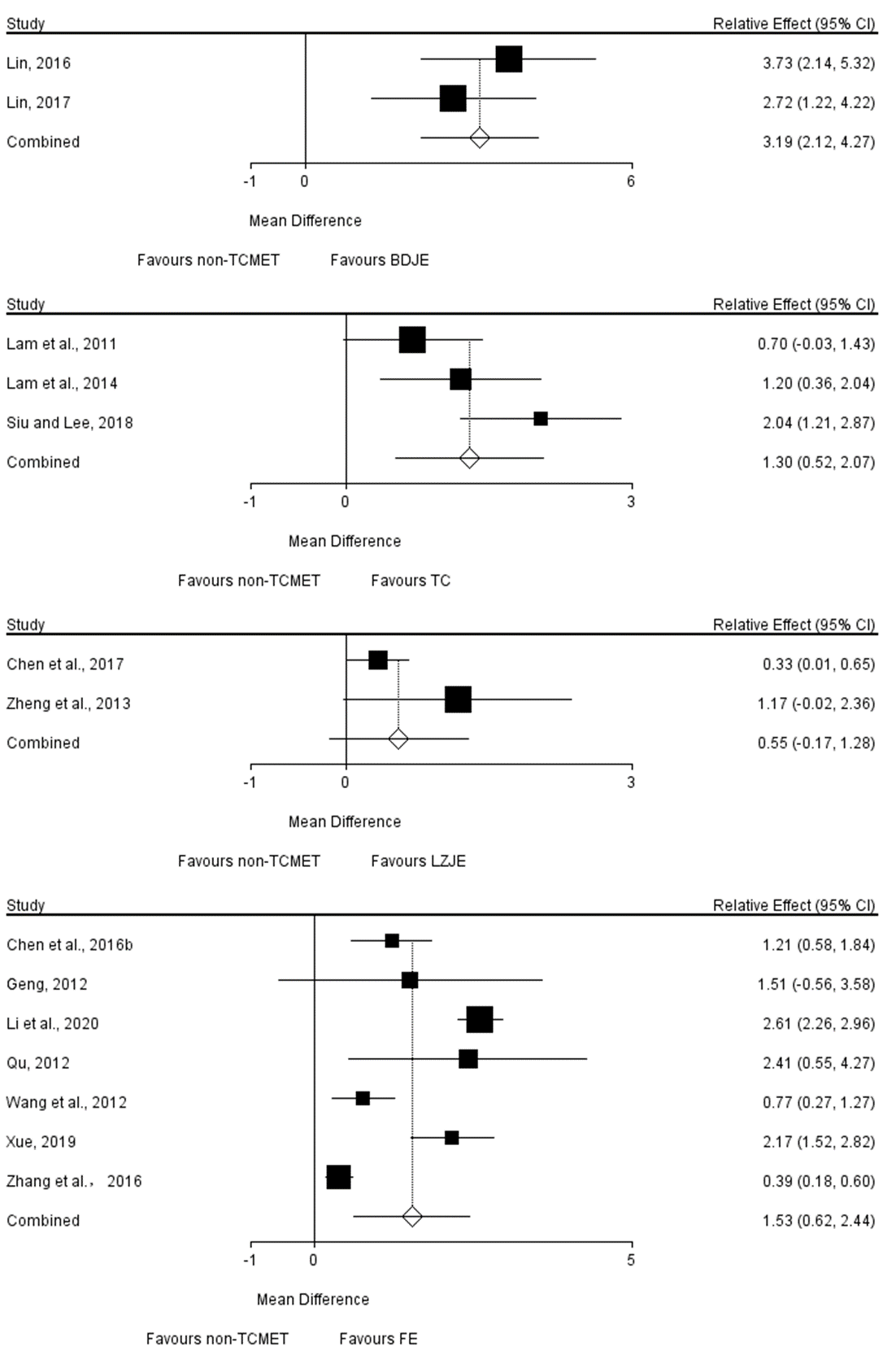


TCMET, traditional Chinese medicine exercise therapy; BDJE, Baduanjin exercise; TC, Tai Chi; LZJE, Liuzijue exercise; FE, finger exercise.

MoCA


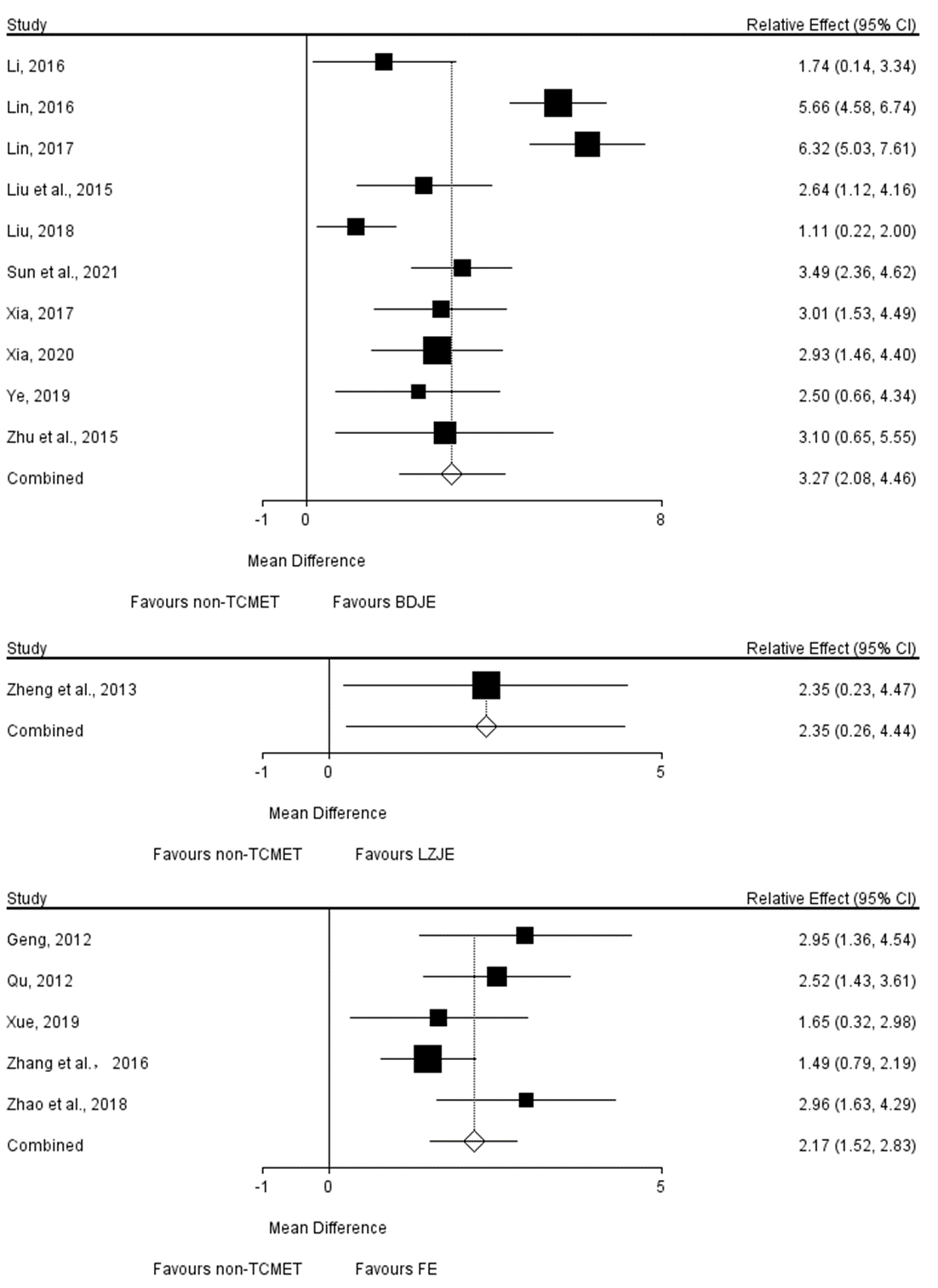


TCMET, traditional Chinese medicine exercise therapy; BDJE, Baduanjin exercise; LZJE, Liuzijue exercise; FE, finger exercise.

ADL


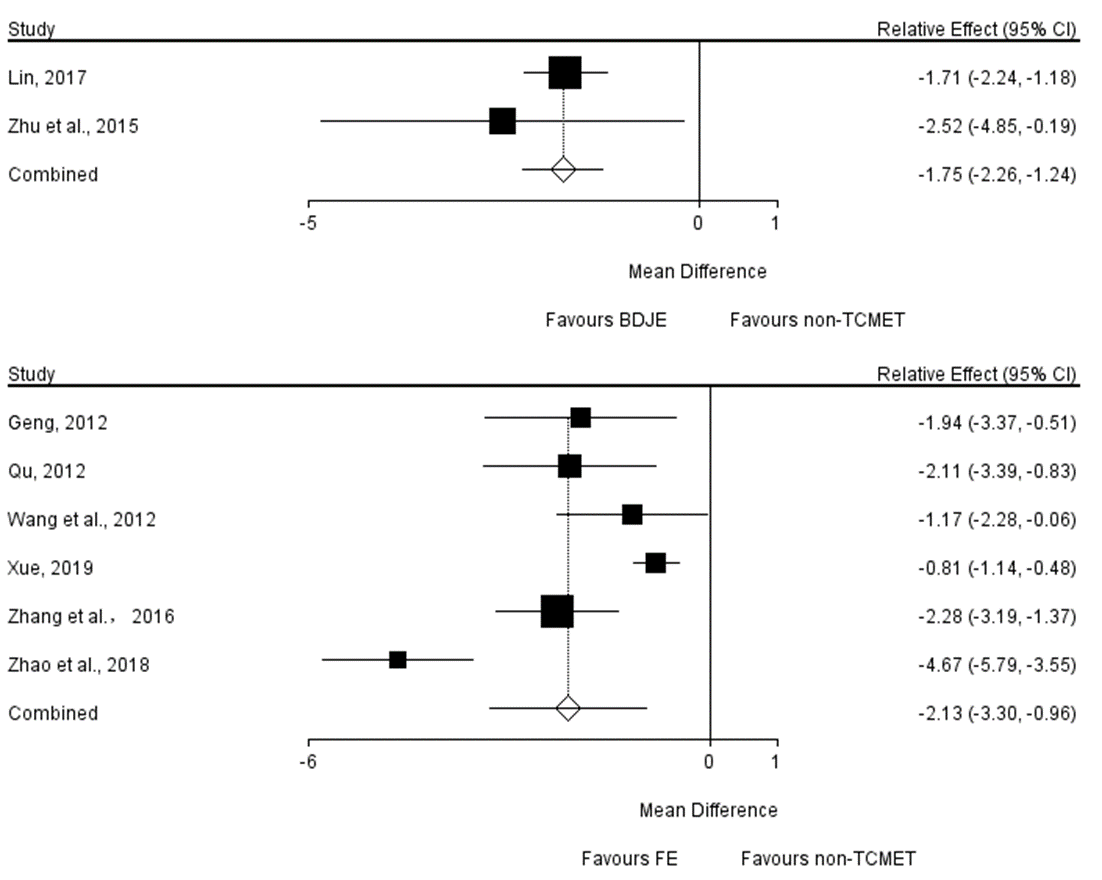


TCMET, traditional Chinese medicine exercise therapy; BDJE, Baduanjin exercise; FE, finger exercise.
